# Some Observations on the Pathology and Prevailing Diseases of Warm Climates

**Published:** 1804-02-01

**Authors:** A. Pearson

**Affiliations:** Member of the London College of Surgeons, and Surgeon in the Service of the East India Company


*Some* OBSERVATIONS *on the* PATHOLOGY *and* PREVAILING DISEASES *of* WARM CLIMATES; *by* A. PEARSON, *Member of the London College of Surgeons, and Surgeon in the Service of the East India Company.*

BEFORE proceeding to consider the diseases which are most prevalent in warm climates, it may be useful to take such a view of the state of the human frame in cold and in hot climates, and the changes induced by a removal from the one to the other, as our limited knowledge of the animal œconomy, and the action of external powers upon it, will allow of.

Among the inhabitants of the more temperate and colder regions of the earth, the natural constitution is that of muscular irritability and promptitude to action; the nervous mobility is not so great, the influence of that power being more equable, steady, and moderate. The solids are denser; of this there is a remarkable proof drawn from the fact, that between an European and an Hindoo or Chinese of apparently equal bulk, the difference of weight is very great, the former being much heavier. The blood contains a greater a greater proportion of crassamentum, and there is also a greater quantity of the circulating mass. The cuticular discharge and the biliary secretion are in smaller quantity; the power of absorption is greater, especially its action in the renewal of the solid parts of the body. The use of animal food and of fermented liquors is more indulged in, and with greater impunity. Such is the natural and the general constitution in cold climates; but adventitious modes of life alter that in very many individuals. Peculiar habits and employments, luxury and sensual gratifications, often induce the temperament of increased mobility and diminished tonic power in them and their progeny, which is in a great measure the same with that which is impressed by hot climates.

In changing from the cold to the hot climate, those effects are produced upon the frame which might be expected to arise from a constant and powerful influence acting upon an accumulated excitability, which heretofore had been accustomed to be obedient to the impression of less powerful stimuli. The circulation is increased and hurried, the respiration becomes more frequent; lassitude and languor are felt, with inaptitude to the customary employments and exertions; there is a greater susceptibility to nervous impressions, and the dispositions are more irritable; all the secretions are increased, the cutaneous and biliary peculiarly so, and the latter is also changed in its qualities, becoming more acrid. In the increase of these Nature has implanted a powerful remedy for the evils which would otherwise ensue, as, in the change taking place in the act of secretion, the fluids produced combine with a greater portion of the heat, and the temperature does not exceed 96° of Fahrenheit, whatever may be that of the surrounding atmosphere. There is also, for the time, an increased action of the absorbents, manifested by the disposition to costiveness.

In this first state, then, diseases of inflammation and of excitement will be produced, and are found to occur. A continued exposure to the effects of heat at length induces a different state of the frame. From the stimulus impressed being stronger, and from the actions of the vessels of the skin and system being greater than in a due proportion to the strength of the vital powers, or to the degree in which the muscular irritability is renewed by the actions of the system, the ultimate effect is relaxation of the fibre, and an irregular or weakened performance of the functions of the different organs. The law of the œconomy is fulfilled, filled, by which it appears that as debility increases, nervous mobility becomes greater also. Where the effects of climate are aided by the abuse of spirituous or fermented liquors, this irritability of frame is increased to a very great degree, and extends itself to the dispositions of the mind.

It may be of some use in illustrating the effects of climate, to advert to those of excessive stimulation from inebriety frequently repeated; and though, as will be shown afterwards, the analogy between the two is not complete, there is a considerable similarity in the state of constitution induced by both. Muscular action is by repetition strengthened, and more readily performed; that of the organs of sense or power of receiving sensual impressions is by the same means diminished. The pleasurable feelings of intoxication, as produced by the application of the liquor to nerves of the organ of taste, by the increased actions and secretions of the vessels and viscera, or by those perhaps of the sensorium itself, by combinations of the memory and imagination, are at length exhausted, and not to be again called forth; the muscular coats of the vessels are more readily induced to act, but their action, though rapid, is enfeebled, desultory, and easily interrupted.—Sometimes it is stopped, or nearly so; but this happens most readily in the lacteals and absorbent lymphatics, then giving rise to atrophy. The digestion is impaired, and the appetite irregular; the biliary secretion is at times suspended, at others increased, and probably vitiated in its qualities: the gastric secretion is most frequently vitiated. A similar libration betwixt increased and suspended action takes place in all the functions; in those also of the sensorium, on which the state of mind depends, there is an obtuseness of perception with regard to objects of external and internal sense, or to those which interest the generality of mankind, with a frequent occnrrence of violent emotions from slight causes. A degree of fever becomes habitual, there is a sinking in of the abdomen, wasting of muscular substance, chronical inflammation and scirrhus of the glandular viscera, and painful sensation in parts where no sensation ought to be felt.

Such a state of health being brought on by residence in hot climates, and also being frequently met with in our own, occasioned by excessive stimulation from other causes than heat, leaves us at a loss how much to ascribe to the effect of a high temperature; the more so, that few reside in these countries without combining the application of of both these hurtful powers. When the constitution has not been injured by excess in the individual, or the effects of it entailed by his progenitors, it is probable that the order of things, which has rendered an intercourse between different climates necessary, has in the œconomy implanted the power of making the change without a sacrifice of the blessings of healthful existence. We find accordingly, that observing a regimen nutritious but not stimulating, and other attentions to the juvantia and lædentia, enable many individuals to suffer it without much injury; while, perhaps, nothing can account for the Orientals having existed so long as nations, to a certain degree civilized, and their having made considerable progress in the arts of life, and even in science, but that an attention to such observances has been imposed upon them, and maintained by the tenets of their religion. It is certain that, in general, a few generations of Europeans terminate that race, and that their mental and moral existence is not even so long protracted; at least, an attention to what has happened among the nations who, in their turns, have had the dominion of the East, would suggest that inference. The density and irritability of the muscular solid, the firmness of mind connected with that, and the advantages of higher civilization, give the Europeans many advantages, which are rendered more permanent by the influence of habit; but their descendants could not retain this pre-eminence, were it not for recruits from the more northern regions.

It will perhaps be easier to specify the precautions to be observed in making the change of climate, than to point out their modus operandi, or the exact method in which the change of climate renders them necessary, and the rules for preserving health will vary according to the different constitutions, periods and habits of life; I will however take for granted that there is nothing peculiar in these respects.

In the first change from a cold to a hot climate it was formerly the practice to bleed indiscriminately; it is now perhaps too generally omitted, as it might be often employed to obviate or remove disease arising from inflammatory congestion. Purging has also been recommended for universal adoption; and when we reflect that the constitution both admits and requires this evacuation more frequently in warm than in cold climates, and bears it better, its utility will be found as probable as experience proves it to be. The neutral salts have generally been prescribed, and these are certainly of the most universal application and use

Inf. sennæ et tamarind. p. rhei. et kali tart. separately or combined, ℈j to ʒß of the former and ʒj to ʒij. of the latter, will be best for frequent use; occasionally, four or five grains of calomel may be taken with much advantage from its effect in stimulating the mucous or biliary excretories, when some of the laxatives above specified ought to be given next morning; the day on which any of these remedies are given ought to be one of peculiar moderation, and dilution with barley-water or rice gruel attended to. In bilious temperaments, where there is a predisposition to hepatic congestion, or where there is any apprehension of the typhus icterodes, it will frequently be found a measure of useful precaution to rub a drachm of mercurial ointment into the side, or to take four of the pil. hydrargyri daily for a week, so as to induce a slight mercurial action in the system.

With regard to the use of tonics, or antiseptics, the indications for employing them, and their utility, are much less than is generally supposed. The feeling of debility is often fallacious, and produced by the organs being overloaded, or a biliary absorption; and the effect of heat in accelerating putrefaction is not applicable to the living œconomy: on the contrary, by leading to greater personal cleanliness and circulation of air, those symptoms denominated putrescent are more rarely met with. The simply bitter medicines are of doubtful safety in every climate, and there is no doubt that an habitual use of them ought to be avoided; when given, it should be at intervals, tonics of the astringent kind requiring to be accompanied with the use of laxative medicines. The food ought, in quantity and quality, to suffice for the purposes of nutrition, and of supporting the healthful action of the system; and the times of meals ought probably to be regulated by former habits, although these are different from what are generally adopted by the natives of warm climates. For breakfast, bread and little butter with tea; bread and soft egg, or the yolk only; rice and fish. Our customs have rendered tea too prevailing an article of regimen. Fruit ought to be used at first sparingly, and in the forenoon. For dinner, soups or broths, provided there is no idiosyncrasy or dyspeptic affection to forbid their use; a moderate portion of well dressed food and vegetables. The principal caution to be observed is, of not exceeding in quantity, or taking in so much as to burthen the stomach. For drink, in general water only. Wine is the least exceptionable of stimulating drinks, but unnecessary and hurtful [illegible] the young young and plethoric; and when the valetudinary have recourse to it, it ought to be in quantity only sufficient to occasion action of the powers, without endangering a subsequent collapse. Malt liquors are peculiarly injurious to those who are predisposed to affections of the liver, and of the biliary and mucous secretions; and spirits, however diluted, seem to have a most injurious effect on the power and activity of the gastric secretion. Melted butter, pastry, &c. are peculiarly objectionable.

If the dinner is late, supper ought to be totally omitted. Sleep ought to be taken early, for seven or eight hours, on a hair mattress, and in an airy place, but not exposed to currents of air: and such ought to be peculiarly guarded against where land winds prevail, or there is an exposure to marsh effluvia. The person ought to rise with the dawn, and at that time, if his situation permits it, to take horse exercise. Cold bathing, when admissible, is an excellent preservative against the effects of heat; and where there are no habits giving rise to plethora and visceral congestion, it ought to be had recourse to daily. To avoid much exercise in, or exposure to the rays of the sun is an injunction of obvious propriety and use; but were due attention paid to other cicumstances, this might be obeyed with a latitude arising from the avocations and employments of every one. Where the frame requires it, a short sleep after dinner may be indulged in, taking care to loosen whatever may bind or obstruct the circulation. In the cool of the evening, horse exercise may be again had recourse to. The mind ought to be at ease; where that is burthened, the disposition to many tropical diseases is increased; hepatic affections are most liable to attack those who suffer from sorrow, anxiety, or disppointment.

[ To be continued. ]

